# Epigenetic rather than genetic factors may explain phenotypic divergence between coastal populations of diploid and tetraploid *Limonium* spp. (*Plumbaginaceae*) in Portugal

**DOI:** 10.1186/1471-2229-13-205

**Published:** 2013-12-06

**Authors:** Ana Sofia Róis, Carlos M Rodríguez López, Ana Cortinhas, Matthias Erben, Dalila Espírito-Santo, Michael J Wilkinson, Ana D Caperta

**Affiliations:** 1 Plant Diversity and Conservation Group, Centro de Botânica Aplicada à Agricultura (CBAA), Instituto Superior de Agronomia (ISA), Universidade de Lisboa, Tapada da Ajuda, 1349-017 Lisboa, Portugal; 2 Plant Genomics Centre, School of Agriculture, Food and Wine, Faculty of Sciences, University of Adelaide, Waite Campus, PMB1, Glen Osmond, SA 5064 Australia; 3 Section Biodiversity Research & Systematic Botany, Maximilian University of Munich, Munich, Germany; 4 Research Network in Biodiversity and Evolutionary Biology (InBIO), ISA, Universidade de Lisboa, Tapada da Ajuda, 1349-017 Lisboa, Portugal

**Keywords:** *Limonium*, Epigenetic variation, Genetic variation, MSAPs, Polyploidy, Ploidy diagnosis

## Abstract

**Background:**

The genus *Limonium* Miller comprises annual and perennial halophytes that can produce sexual and/or asexual seeds (apomixis). Genetic and epigenetic (DNA methylation) variation patterns were investigated in populations of three phenotypically similar putative sexual diploid species (*L. nydeggeri, L. ovalifolium*, *L. lanceolatum*), one sexual tetraploid species (*L. vulgare*) and two apomict tetraploid species thought to be related (*L. dodartii, L. multiflorum*). The extent of morphological differentiation between these species was assessed using ten diagnostic morphometric characters.

**Results:**

A discriminant analysis using the morphometric variables reliably assigns individuals into their respective species groups. We found that only modest genetic and epigenetic differentiation was revealed between species by Methylation Sensitive Amplification Polymorphism (MSAP). However, whilst there was little separation possible between ploidy levels on the basis of genetic profiles, there was clear and pronounced interploidy discrimination on the basis of epigenetic profiles. Here we investigate the relative contribution of genetic and epigenetic factors in explaining the complex phenotypic variability seen in problematic taxonomic groups such as *Limonium* that operate both apomixis and sexual modes of reproduction.

**Conclusions:**

Our results suggest that epigenetic variation might be one of the drivers of the phenotypic divergence between diploid and tetraploid *taxa* and discuss that intergenome silencing offers a plausible mechanistic explanation for the observed phenotypic divergence between these microspecies. These results also suggest that epigenetic profiling offer an additional tool to infer ploidy level in stored specimens and that stable epigenetic change may play an important role in apomict evolution and species recognition.

## Background

DNA sequence divergence clearly plays a leading role in shaping the phenotypic variation observed between most taxa e.g. [1,2] but does not explain all forms of adaptive phenotypic differentiation [3-5]. Epigenetic modifications of DNA and histones, the core components of chromatin are able to influence the expression of the underlying genes and so phenotype [6]. Of these epigenetic mechanisms, the best studied and the one to show more examples of transgenerational stability is cytosine DNA methylation [6,7]. Evidence has also been found suggesting that heritable phenotypic variation observed in natural populations can be due to stable epigenetic variation, even in the absence of genetic variation and could play a role in plant adaptation and evolution [5,8-10]. The scope for epigenetic mechanisms driving morphological differentiation is perhaps best illustrated when genetic variation is lacking. Understanding the relative importance of genetic and epigenetic sources of phenotypic variation where both systems are operating is therefore attracting increasing interest. For example, *Laguncularia racemosa* is a mangrove plant species that shows low genetic variability but in populations from distinct, nearby habitats, cytosine methylation variation among individuals correlates more closely with environmental variation than does genetic variation [11]. In the perennial *Viola cazorlensis*, cytosine methylation patterns were found to be partitioned and positively correlated with adaptive genetic variation [12]. Also, in populations of individuals with reduced or negligible genetic variation such as those of triploid asexual dandelion lineages (apomixis; diplospory), changes in genomic methylation patterns are found between individuals [13].

The genus *Limonium* Miller (sea-lavenders; *Plumbaginaceae*) has long been recognized to have a history of recurrent hybridization and polyploidization, and comprises 150 [14] to 350 taxa recognized across coastal, steppe and desert regions (e.g. [15,16]). This wide range is due to the description of new taxa, mainly microspecies from geographically restricted areas. In this genus*,* a sporophytic self-incompatibility system is linked with pollen-stigma dimorphisms, A-pollen type grains germinate on papillose stigmas and B-pollen type germinate in *cob*-like stigmas, while the complementary combinations produce no successful fertilization [17,18]. Most sexual species of *Limonium* usually have a dimorphic self-incompatibility system (both pollen and stigmas are dimorphic) while agamospermous species are generally monomorphic and have monomorphic populations [14,19]. Determination of these characters in individuals from natural and/or experimental populations has since long been used as an indirect method for estimation of each species reproduction mode [14,17,18]. The high number of polyploid taxa has been explained to be a consequence of this self-incompatibility system and the ability of polyploid hybrids to produce seeds asexually via apomixis (agamospermy; asexual seed formation) [14,17-20]. *Ixeris*-type embryo sacs with non-haploid eggs are found in triploid (2*n* = 3x = 27) *Statice oleaefolia* var. *confusa*[20]. In triploid and tetraploid agamospermous species of the *L. binervosum* (G. E. Sm.) Salmon group diplospory followed by parthenogenesis is reported [21,22]. Molecular phylogenetic studies have tried to resolve the taxonomic complexity within this genus in a global perspective using nuclear DNA sequence information [23] and plastid DNA [24-26].

In Continental Portugal about 15 *Limonium* species have been recognized with ecological importance for plant communities of the Atlantic and Mediterranean coastlines [15,27]. Among these, the *L. ovalifolium* complex consists a group of three sexual diploids (2*n* = 2x =16): *L. ovalifolium* (Poir.) O. Kuntze, *L. nydeggeri* Erben and *L. lanceolatum* (Hoffmanns & Link) Franco [28]. The first species has a broader distribution including several Sites of Community Importance (SCI) for the Mediterranean biogeographical regions [29] in the West (Estremadura), Southwest Alentejo and Algarve coastlines [15,30]. Conversely, the Lusitania endemic *L. nydeggeri* and *L. lanceolatum* have more restricted distributions; the former is restricted to West and Southwest Atlantic sea-cliffs whereas the latter is found in the Southwest and South coastlines [28,30]. *Limonium* tetraploid taxa include among others, the Lusitania endemic apomict, *L. multiflorum* Erben [14,15] which exhibits both tetraploid and aneuploid tetraploid cytotypes (2*n* = 4x = 35 - [14]; 2*n* = 4x = 32, 34, 35, 36 – [31]) and the aneuploid tetraploid apomict *L. dodartii* (Girad) O. Kuntze (2*n* = 4x = 35) which most frequently grow on maritime cliffs in the province of Estremadura. A third tetraploid, *L. vulgare* (2*n* = 4x = 36), a sexual species [19,32,33], grows in salt marshes [15,30].

Dominant genetic markers, such as those generated by Amplified Fragment Length Polymorphism (AFLP), are valuable for assessing genetic diversity within and between populations [34] and for inferring taxon differentiation [35], especially in species for which codominant markers are unavailable. Some recent publications have added data on natural epigenetic variation in animal and plant species by sampling cytosine methylation using Methylation-Sensitive Amplification Polymorphism (MSAP) technology [36-38], a modification to the original AFLP protocol that compares product profiles generated by methylation-sensitive/insensitive isoschizomers. Central to the technique is the differential behavior of the two isoschizomer restriction enzymes (*Hpa*II and *Msp*I) in the presence of cytosine methylation in the CCGG context. *Hpa*II is inactive if one or both cytosines are methylated at both DNA strands, but cleaves when one or both cytosines are methylated in only one strand. *Msp*I, by contrast, cleaves C5mCGG but not 5mCCGG. Comparison of the profiles generated by each enzyme from each individual allows the assessment of the methylation state of the restriction sites and so provides a relative comparison of genetic and epigenetic variability. Several reports suggest that only methylation marks in the CG context can be transmitted between generations and so have potential for stable adaptive significance [39]. Furthermore, recent data shows that although non-CG methylation can be inherited, only inherited CG methylation is inversely correlated with gene expression [40]. Since only *Hpa*II is affected by methylation of this kind, for simplicity, in this study we refer to profiles from this enzyme as epigenetic (meaning potentially transgenerationally stable and epigenetic) whereas *Msp*I is insensitive in this sense and so can only detect transgenerationally relevant genetic variation. The focus of this study was to therefore to use MSAP analysis as the primary tool in comparing the extent to which genetic and epigenetic diversity in natural populations of diploid and tetraploid *Limonium* species correlate with species identity and ploidy.

## Results

### Morphological differentiation between diploid and tetraploid species

Herbarium specimens of the diploid species *L. ovalifolium*, *L. nydeggeri* and *L. lanceolatum* and of the tetraploid species *L. dodartii*, *L. multiflorum* and *L. vulgare* obtained from individuals sampled in natural populations were used for morphometric measurements. Ten diagnostic characters were selected based on an exhaustive review of *Limonium* species in Southwest Europe by Erben [14,15], and on previous biometric studies in the *Limonium* genus [33,41,42].

**Figure 1:**
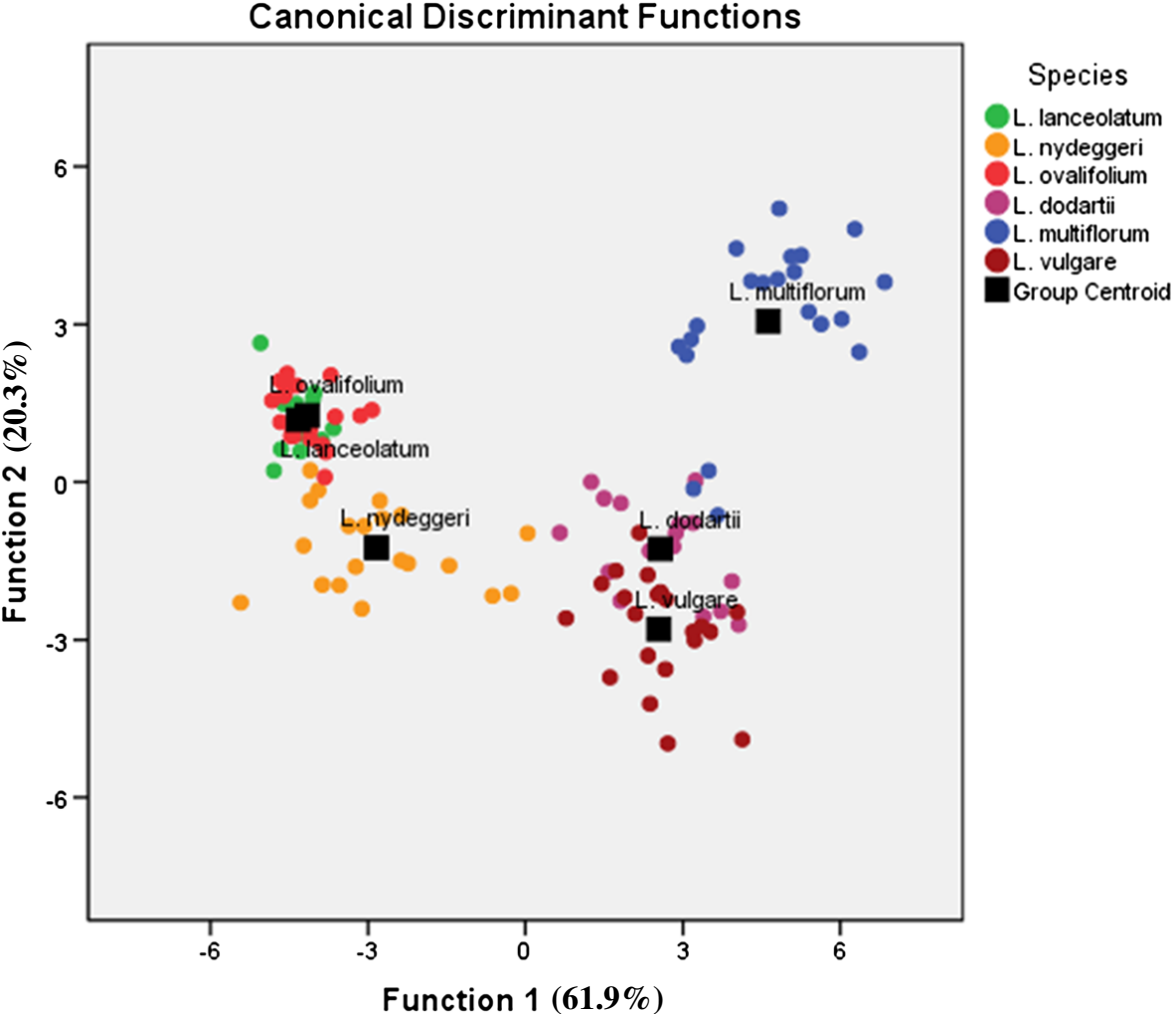
**Discriminant function analyses of morphometric data with predefined diploid and tetraploid *Limonium *species.** Individuals from each diploid (*L. lanceolatum, L. nydeggeri, L. ovalifolium*) or tetraploid (*L. dodartii, L. multiflorum* and *L. vulgare*) species are represented by colour symbols. Each species centroid is represented by filled squares. Percentages of total variance explained by the functions are given in parentheses.

**Figure 2:**
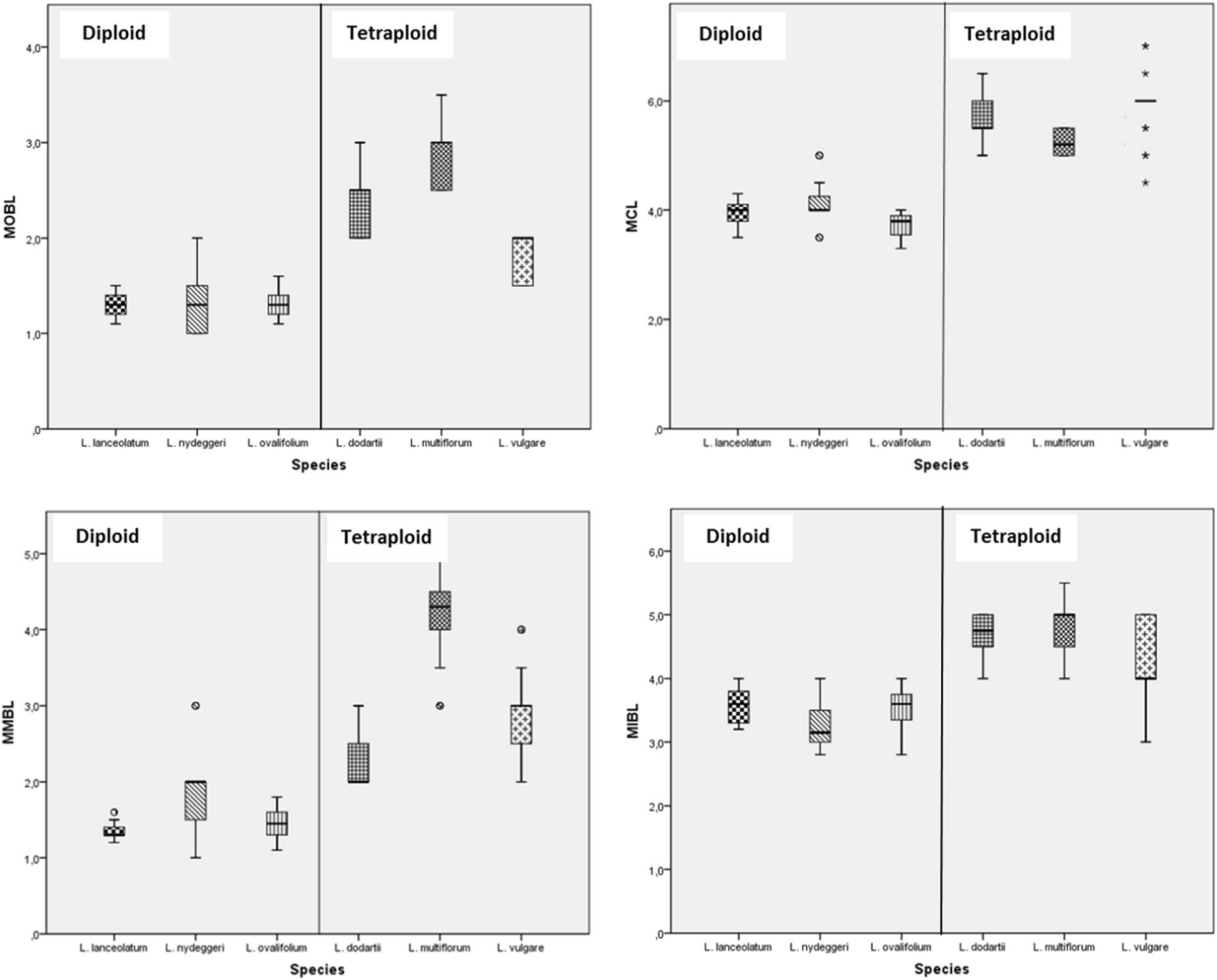
**Box plots of significant characters that discriminate diploid from tetraploid *Limonium *species.** The box from diploids *L. nydeggeri, L. ovalifolium*, *L. lanceolatum*, and tetraploids *L. dodartii, L. multiflorum*, *L. vulgare* show the twenty-fifth and seventy-fifth percentile ranges and the median; circles and asterisks are outliers (cases with values between 1.5 and 3 box lengths from the upper or lower edge of the box). MOBL - Maximum outer bract length; MCL - Maximum calyx length; MMBL - Maximum middle bract length; and MIBL - Maximum inner bract length.

Only one of the ten morphological variables measured from representatives of the six species fitted a normal distribution, Maximum inner bract length (MIBL), whilst the other nine failed to do so, even after a logarithmic transformation. The remaining analyses were therefore performed using the original (untransformed) values (Additional file 1). Canonical discriminant analysis (CDA) of the morphological variables accounted for most of the variation (82.2% in the first two dimensions, comprising 61.9 and 20.3%, respectively) and correctly assigned individuals to species in 92.7% of cases (n = 110; Figure 1; Table 1). However, a small number of intermediate or ambivalent specimens were encountered (Wilks’ lambda = 0.002, χ ^2^ = 650.259 > χ^2^_0.05;50_ = 67.505, P < 0.001). The first axis distinguished diploid from tetraploid species through the following characters: Maximum outer bract length (MOBL), Maximum calyx length (MCL), Maximum middle bract length (MMBL) and MIBL (Table 2; Figure 2). The second axis separated *L. nydeggeri* from the other diploid species and *L. multiflorum* from the other tetraploid species by the following characters: Maximum middle bract width (MMBW), Maximum outer bract width (MOBW) and Maximum inner bract width (MIBW). Thus, these features were largely responsible for separation of species sharing the same ploidy level.

**Table 1:** **Summary of the discriminant analysis of six predefined diploid and tetraploid *Limonium *** **species**

**Species**			**Predicted group membership-classification results**						**Total**
			***L. lanceolatum***	***L. nydeggeri***	***L. ovalifolium***	***L. dodartii***	***L. multiflorum***	***L. vulgare***	
**Original**	**Count**	***L. lanceolatum***	10	0	1	0	0	0	11
		***L. nydeggeri***	0	20	0	0	0	0	20
		***L. ovalifolium***	3	0	18	0	0	0	21
		***L. dodartii***	0	0	0	16	0	0	16
		***L. multiflorum***	0	0	0	1	18	2	21
		***L. vulgare***	0	0	0	1	0	20	21
	**%**	***L. lanceolatum***	90.9	0	9.1	0	0	0	100
		***L. nydeggeri***	0	100	0	0	0	0	100
		***L. ovalifolium***	14.3	0	85.7	0	0	0	100
		***L. dodartii***	0	0	0	100	0	0	100
		***L. multiflorum***	0	0	0	4.8	85.7	9.5	100
		***L. vulgare***	0	0	0	4.8	0	95.2	100

Individuals from each diploid (*L. lanceolatum, L. nydeggeri, L. ovalifolium*) or tetraploid (*L. dodartii, L. multiflorum* and *L. vulgare*) species are classified.

**Table 2:** Pooled within-groups correlations between discriminating variables and standardized canonical discriminant functions of morphological characters

	**Function**				
	**1**	**2**	**3**	**4**	**5**
**MOBL**^**1**^	0.586^*^	0.414	-0.047	0.37	0.311
**MCL**	0.569^*^	-0.44	-0.203	-0.165	-0.123
**MMBL**	0.512^*^	0.298	0.336	-0.434	0.032
**MIBL**	0.350^*^	0.144	-0.203	0.193	0.021
**MMBW**	0.405	0.537^*^	0.306	-0.348	0.151
**MOBW**	0.256	0.527^*^	-0.045	0.318	0.343
**MIBW**	-0.02	0.442^*^	0.01	0.223	-0139
**MNSC**	-0.334	0.288	-0.603	-0.372	0.418^*^
**MSL**	-0.304	-0.08	0.39	0.195	0.656^*^
**MNFS**	-0.005	0.174	0.117	0.075	-0.493

Variables were ordered by absolute size of correlation within function.

^1^MSL, Maximum spike length; MNSC, Maximum number of spikelets per cm; MNFS, Maximum number of florets per spikelet; MOBL, Maximum outer bract length; MOBW, Maximum outer bract width; MMBL, Maximum middle bract length; MMBW, Maximum middle bract width; MIBW, Maximum inner bract width; MCL, Maximum calyx length.

*Largest absolute correlation between each variable in respective function.

### MSAP profiles analysis

**Figure 3:**
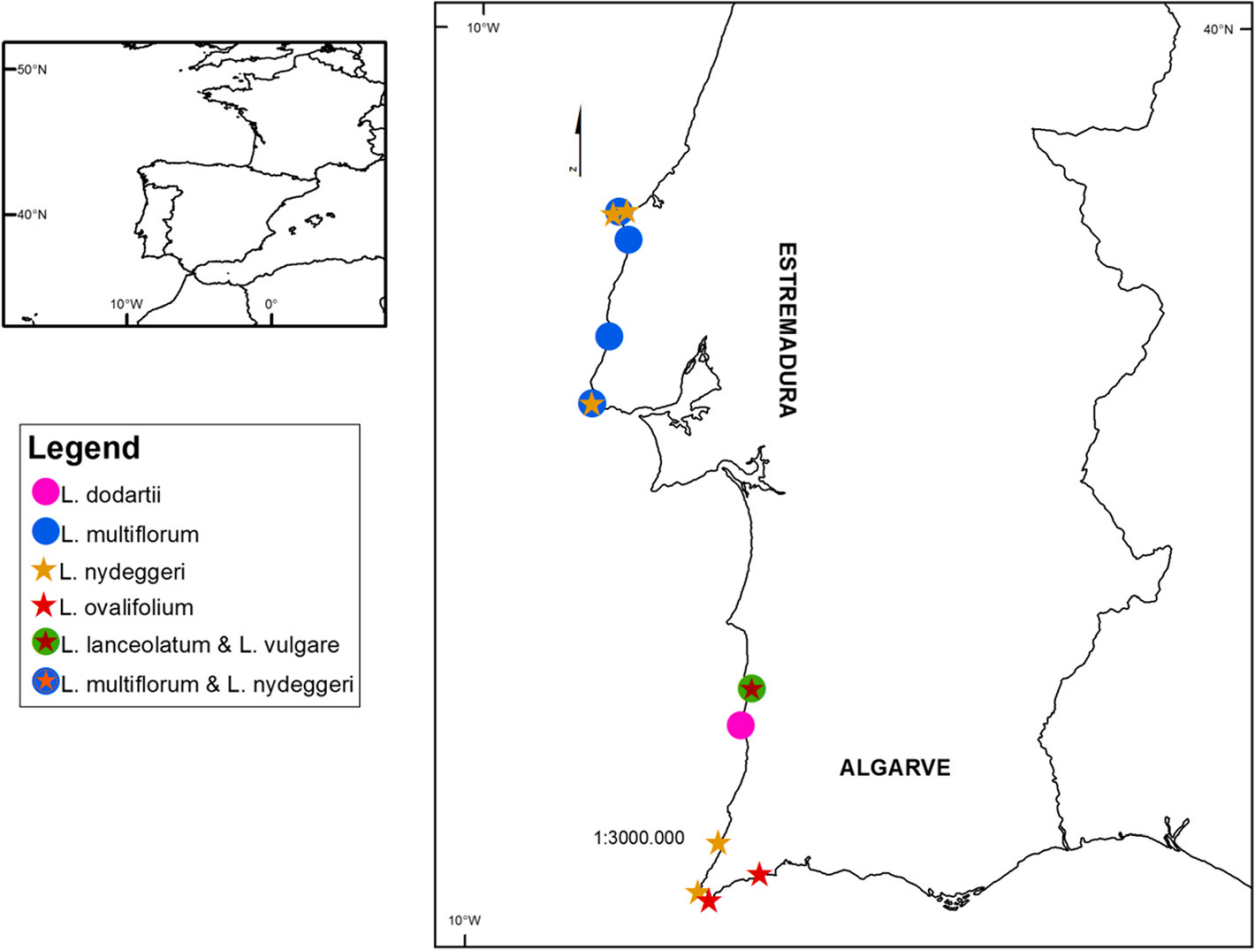
**Diploid and tetraploid *Limonium *populations sampled in continental Portugal in Sites of Community Interest.** Diploid species (*L. lanceolatum, L. nydeggeri* and *L. ovalifolium*) are represented by full stars and tetraploid species (*L. dodartii*, *L. multiflorum* and *L. vulgare*) are represented by full circles. Specimens were sampled in Estremadura (PTCON0056: Baleal, Papoa, Nossa Sra dos Remédios, Vale dos Frades, Foz do Lizandro; PTCON0008: Cabo Raso), Alentejo (PTCON0012: Vila Nova de Milfontes, Pontal da Carrapateira), and in Algarve (PTCON0012: Cabo de Sagres; Praia da Luz) provinces.

Genetic-epigenetic analyses were performed on 125 individuals selected from natural populations within designated SCI(s) (Figure 3; Table 3). The two MSAP primer combinations applied to all samples yielded 835 scorable fragments comprising 792 from *Msp*I and 778 from *Hpa*II, 92.78% and 95.36% respectively, were polymorphic (i.e. not present in all the analysed samples/replicates when restricted with one of the isoschizomers). Overall reproducibility between biological replicates was 83% and 85% for primer combinations E1/H1 and E1/H3 respectively. The methylation insensitive (genetic variation) profiles showed only very slightly higher concordance among replicates (83.6% and 85.7%) than did the methylation-sensitive (epigenetic variation) profiles (82.4% and 84.3%). Technical reproducibility of the MSAP technique revealed between 92-95% band concordances (data not shown), indicating that the higher variability between independent DNA extractions probably arises from variation between tissues and cell mixtures used in the DNA extraction.

**Table 3:** **Collection data of fourteen populations of *Limonium *** **species included in the MSAP analyses**

**Ploidy level**	**Species**	Site location collector^*^	**Geographical coordinates latitude/longitude**	**N**
**Diploid**	***L. lanceolatum***	Odemira, Vila Nova de MilFontes, ADC, APP, ASR	37,727756/-8,770931	10
		Peniche, Ilha do Baleal, ADC, APP, ASR	39,378919/-9,340983	9
		Peniche, Nossa Sr^a^ dos Remedios, ADC, APP, ASR	39,369906/-9,395731	7
	***L. nydeggeri***	Cascais, Cabo Raso, ADC, APP, ASR	38,710039/-9,485883	7
		Aljezur, Pontal de Carrapateira, ADC, APP, ASR	37,195039/-8,911103	10
		Vila do Bispo, Cabo de São Vicente, ADC ASR	37,002611/-8,996564	10
	***L. ovalifolium***	Vila do Bispo, Cabo de Sagres, ADC, APP, ASR	36,994242/-8,948756	6
		Lagos, Praia da Luz ADC, ASR	37,087442/-8729094	10
**Tetraploid**	***L. dodartii***	Odemira, Cabo Sardão, ADC, APP, ASR	37,598344/-8,818272	10
	***L. multiflorum***	Peniche, Península da Papoa, ADC, APP, ASR	39,374131/-9,377428	6
		Lourinhã, Vale dos Frades, ADC, APP, ASR	39,276506/-9,335839	7
		Mafra, Foz do Lizandro, ADC, APP, ASR	38,941531/-9,415223	9
		Cascais, Cabo Raso, ADC, APP, ASR	38,710039/-9,485883	14
	***L. vulgare***	Odemira, Vila Nova Mil Fontes, ADC, APP, ASR	37,727756/-8770931	10

The geographical location of each population is represented in Figure 3. Geographical coordinates of each population and sampling size (*N*; approximately 10 individuals per population) are included.

*Abbreviations of collectors: ADC, AD Caperta: APP AP Paes; ASR, AS Rois; AC, Ana Cortinhas. Centro de Botânica Aplicada Agricultura, Instituto Superior de Agronomia, Lisboa, Portugal.

Profiles from the tetraploid species (i.e. *L. multiflorum, L. dodartii, L. vulgare*) included a higher number of *Msp*I fragments (genetic profiles) per individual for both primer combinations (Table 4). Conversely, these species contained a lower number of *Hpa*II-generated (methylation-sensitive epigenetic profiles) and fewer fragments per individual than the three diploid species (i.e. *L. nydeggeri, L. ovalifolium, L. lanceolatum*), implying a higher level of genome-wide methylation among tetraploids.

**Table 4:** MSAP fragment number analysis

**Ploidy level**	**Species**	**E1/H1**		**E1/H3**		**Total**	
		***Hpa*** **II**	***Msp*** **I**	***Hpa*** **II**	***Msp*** **I**	***Hpa*** **II**	***Msp*** **I**
**Diploid**	***L. lanceolatum***	52	52	47	38	99	90
	***L. nydeggeri***	50	48	43	33	93	81
	***L. ovalifolium***	48	43	42	28	90	71
**Tetraploid**	***L. dodartii***	37	77	24	56	61	133
	***L multiflorum***	33	59	30	35	63	94
	***L. vulgare***	26	46	20	39	46	85

Average number of MSAP fragments per diploid (*L. lanceolatum, L. nydeggeri, L. ovalifolium*) and tetraploid (*L. dodartii, L. multiflorum* and *L. vulgare*) species obtained using isoschizomers enzymes *Hpa*II (methylation sensitive) and *Msp*I (methylation insensitive) and primer combinations E1/H1 and E1/H3.

### Genetic/epigenetic divergence of diploid and tetraploid species

**Figure 4:**
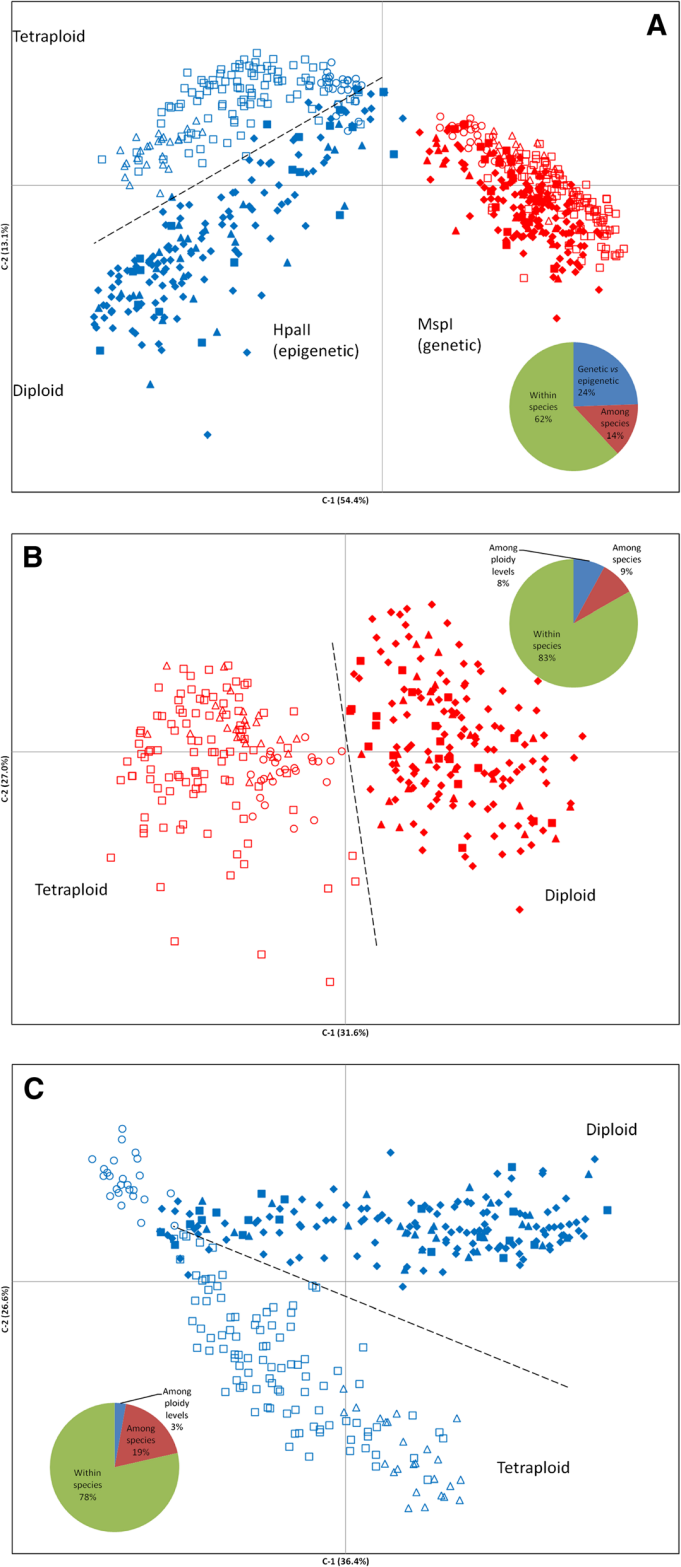
**Principal Coordinate Analysis (PCoA) representing genetic and epigenetic variability in diploid and tetraploid *Limonium *species.** PCoA was based on presence/absence scores of 488 polymorphic loci obtained from MSAP profiles using isoschizomers *Msp*I (methylation insensitive - red symbols in **A** and **B)** or *Hpa*II (methylation sensitive - blue symbols in **A** and **C)** as frequent cutters and amplified with primers (E1/H3). The first two coordinates were extracted and plotted against each other. Percentage of the variability shown by each coordinate is indicated between parentheses. Diploid species are represented by solid symbols (*L. lanceolatum*, triangles; *L. nydeggeri*, rhomboids; *L. ovalifolium*, rectangles) and tetraploid species are represented by empty symbols (*L. dodartii*, triangles; *L. multiflorum*, rectangles; *L. vulgare*, circles).

Principal Coordinate Euclidean Analysis (PCoA) was used to provide an overview of the genetic/epigenetic variability and structure of the studied taxa. Overall, epigenetic profiles (*Hpa*II) provided imperfect but slightly better separation of the taxa than did the genetic profiles (*Msp*I) (Figure 4A; Additional file 2). Genetic data (*Msp*I) alone also largely failed to discriminate within the diploid species and the tetraploid species (Figure 4B; Additional file 2). More remarkably, PCoA plots of the epigenetic *Hpa*II profiles revealed clear separation between the diploid and tetraploid *taxa* (Figure 4C; Additional file 2).

On average, genetic and epigenetic distances between the three diploid species were significantly lower than comparable distances between the tetraploid species (T-test, Genetic distance p < 0.023; epigenetic distance p < 0.0006 (two-tailed test); Additional file 3). Furthermore, calculated epigenetic distances between individuals from different diploid species and between tetraploid species were higher than genetic distances between the same pairings (T-test, p < 0.04 (between diploid species); p < 0.15 (between tetraploid species) (two-tailed test); Additional file 3).

Analysis of genetic/epigenetic variability using Analysis of Molecular Variance (AMOVA) showed that 24% of the total observed variability can be attributed to differences between epigenetic (*Hpa*II) and genetic (*Msp*I) sources of variability (Figure 4A; Additional file 2). Independent analysis of results generated by each enzyme type revealed that differences between diploid and tetraploid samples accounted for 6-8% of the genetic variability and for 3-6% of the epigenetic variability. This compared with a much higher level of within-species variation, which comprised 79-83% and 74-78% of the genetic and epigenetic variability respectively (Figure 4B-C; Additional file 2). Surprisingly, while genetic differences between species accounted for 9-15%, epigenetic differences accounted for 19-20%, suggesting that characterisation of these very closely related species is best served by considering both genetic and epigenetic information rather than genetic information only (Additional file 3).

### Tetraploid species compensate lower genetic variability with higher epigenetic variability

Closer consideration of the genetic and epigenetic variability revealed that all species had more epigenetic than genetic variability. And, the diploid species were more variable (both at genetic and epigenetic level) than were the tetraploid ones (Table 5). However, the difference between genetic and epigenetic variability was higher among the tetraploid species despite containing lower levels of variability overall (Table 5).

**Table 5:** **Genetic and epigenetic variability within diploid and tetraploid *Limonium *** **species**

**Primer**		**Ploidy level: Diploid/Tetraploid**								
		**%PL**			**Sh I**			**He**		
**E1/H1**	**Genetic**	74.45/52.93%			0.183/0.116			0.106/0.066		
	**Epigenetic**	82.23/58.79/%			0.204/0.098			0.118/0.110		
**E1/H1**	**Genetic**	63.32/31.21%			0.104/0.062			0.054/0.035		
	**Epigenetic**	69.67/42.89%			0.128/0.098			0.071/0.058		
**Average**	**Genetic**	68.88/42.072%			0.143/0.089			0.095/0.084		
	**Epigenetic**	75.95/50.84%			0.166/0.139			0.095/0.084		
**Primer**		***L. nydeggeri***			***L. ovalifolium***			***L. lanceolatum***		
		**%PL**			**Sh I**			**He**		
**E1/H1**	**Genetic**	90.29%	0.191	0.107	51.71%	0.132	0.073	80.29%	0.222	0.127
	**Epigenetic**	90.29%	0.194	0.112	61.14%	0.188	0.113	64.86%	0.194	0.118
**E1/H3**	**Genetic**	85.25%	0.103	0.052	34.63%	0.075	0.040	65.16%	0.124	0.064
	**Epigenetic**	85.66%	0.130	0.071	48.16%	0.119	0.066	59.43%	0.132	0.074
**Average**	**Genetic**	87.77%	0.147	0.079	43.17%	0.103	0.056	72.72%	0.173	0.095
	**Epigenetic**	87.97%	0.162	0.092	54.65%	0.154	0.090	62.14%	0.163	0.096
**Primer**		***L. multiflorum***			***L. dodartii***			***L. vulgare***		
		**%PL**			**Sh I**			**He**		
**E1/H1**	**Genetic**	76.86%	0.186	0.110	47.14%	0.130	0.075	35.14%	0.089	0.051
	**Epigenetic**	66.29%	0.119	0.067	42.86%	0.186	0.120	38.29%	0.131	0.082
**E1/H3**	**Genetic**	65.57%	0.094	0.051	22.95%	0.060	0.034	20.90%	0.048	0.027
	**Epigenetic**	49.18%	0.077	0.043	27.05%	0.104	0.067	32.99%	0.092	0.056
**Average**	**Genetic**	71.22%	0.140	0.081	35.05%	0.095	0.055	28.02%	0.069	0.039
	**Epigenetic**	57.73%	0.098	0.055	34.95%	0.0145	0.093	35.64%	0.112	0.69

Calculated Percentage of Polymorphic Loci (%PL), Shannon diversity index (ShI) and Expected Variability (He) – 2*p*q) for diploid (*L. lanceolatum, L. nydeggeri, L. ovalifolium*) and tetraploid (*L. dodartii, L. multiflorum* and *L. vulgare*) species. All values obtained using GenAlex (v.6.4).

**Figure 5:**
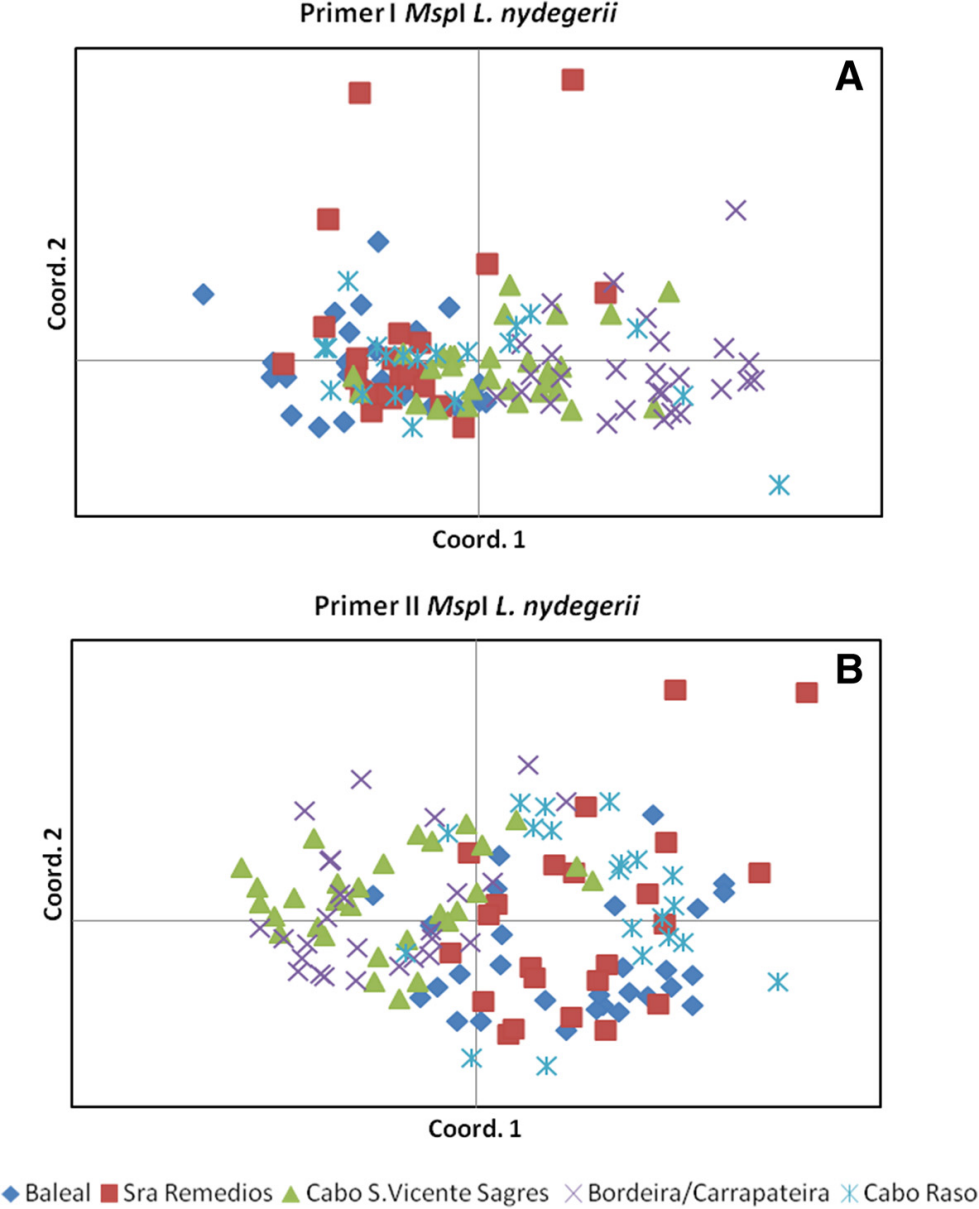
**Principal Coordinate Analysis (PCoA) representing genetic variability in *Limonium nydeggeri *populations.** PCoA was based on presence/absence scores of 488 (primer I, E1/H1) **(A)** and 347 (primer II, E1/H3) **(B)** polymorphic loci obtained from MSAP profiles using isoschizomers *Msp*I (methylation insensitive) or *Hpa*II (methylation sensitive) as frequent cutters. The first two coordinates were extracted and plotted against each other.

PCoA of the genetic profiles revealed no or only weak co-clustering of individuals according to population origin, with the best example being seen among *L. nydeggeri* samples (Figure 5 A-B). No co-clustering was evident from the epigenetic profiles (data not shown). A lack of structuring according to population origin was further supported by AMOVA, with the main component of both genetic (90-97%) and epigenetic variance (86-95%) residing within populations.

**Figure 6:**
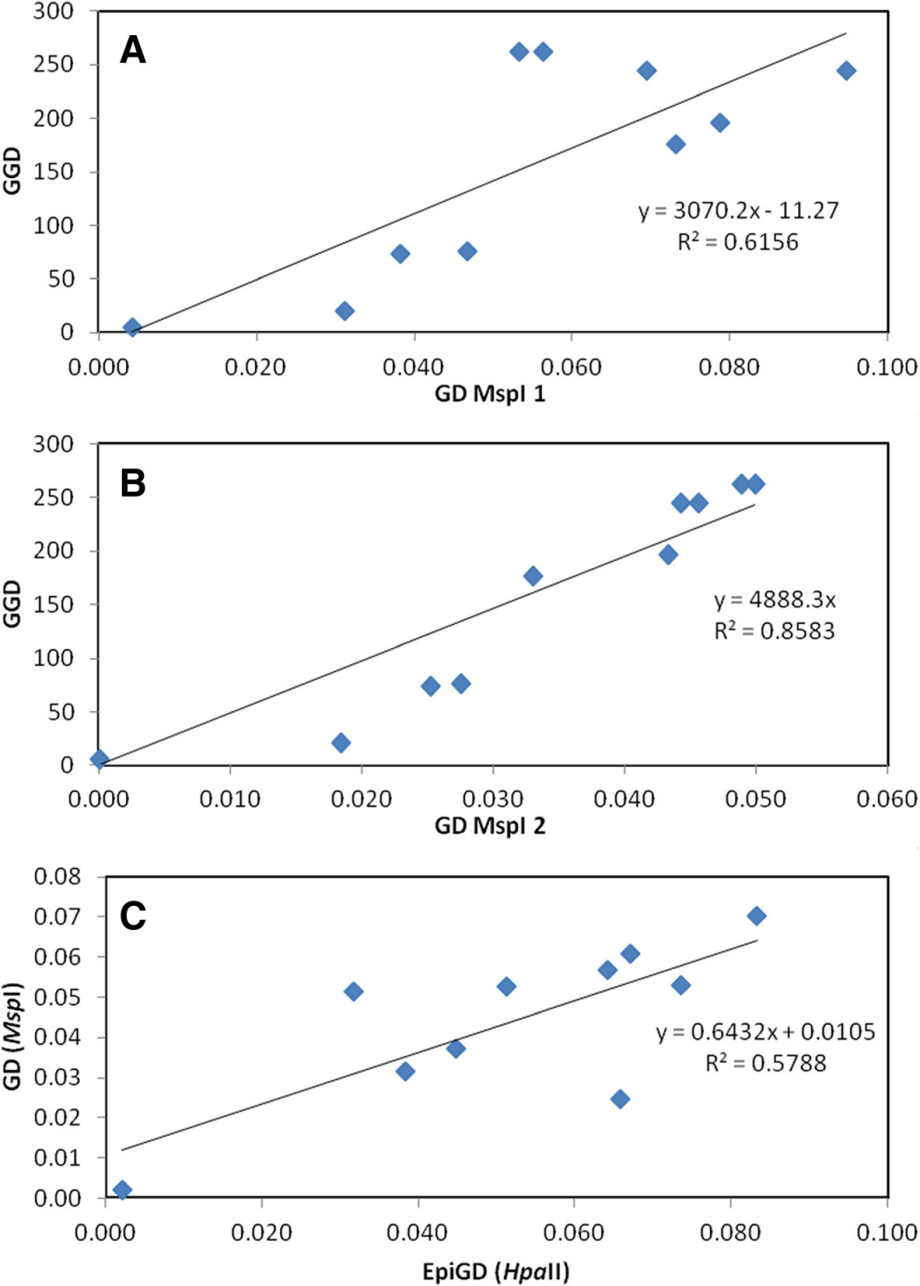
**Correlation between pairwise genetic differentiation (GD, PhiPT) and geographical distance (GGD, in Km) (A-B) and between pairwise genetic and epigenetic differentiation (GD and EpiGD, respectively) (C) between *Limonium nydeggeri *populations.** Mantle tests were based on MSAP data obtained using *HpaII* and primer combinations primer I (E1/H1) **(A)** and primer II (E1/H3) **(B)** and *HpaII* and *MspI* and both primers **(C)**. Shown equations are the linear functions and R^2^ values for each Mantel test. Analysis using 1000 permutation tests showed a significant correlations (A: P < 0.04; B: P < 0.02; C: P < 0.03).

We next sought structuring across a geographic scale. Mantel test analysis revealed a correlation between genetic distances and geographic separation among conspecific populations for *L. nydeggeri* (R^2^ = 0.784, P < 0.03). This correlation was significant for both primer pairs (H1/E1, R^2^ = 0.858, P < 0.02; H1/E3, R^2^ = 0.616, P < 0.04) (Figure 6 A-B). Both primers generated a scatterplot showing a positive and monotonic relationship over all geographic distances of separation. In contrast, *L. multiflorum* populations showed no detectable relationship between genetic distance and geographic distance and large variance in estimates of divergence. None of the studied species showed a significant correlation between epigenetic and geographic distances, with extensive scatter between the plotted samples (data not shown).

Finally, Mantel test analysis revealed a strong significant positive correlation between genetic and epigenetic distances among *L. nydeggeri* populations (H1/E1, R^2^ = 0.800, P < 0.008; H1/E3, R2 = 0.200, P < 0.07 and R^2^ = 0.600, P < 0.005, using the information provided by both primer combinations) (Figure 6 C). Conversely, there was only a weak negative correlation between genetic and epigenetic distances among *L. multiflorum* populations (data not shown).

## Discussion

The effects of hybridization, polyploidy and apomixis have all combined to shape radiation currently seen in *Limonium* species [26,31,43]. This evolutionary model has been used to explain multiple series of complex aggregates of sexual diploid species and asexual polyploid hybrids which are perpetuated through gametophytic apomixis [44-48]. In other plant groups, hybridization and polyploidy have combined to generate genetic and phenotypic complexity in the form of classical polyploidy pillar complexes. In these situations species delimitations typically become blurred at the higher ploidy levels and interploidy discrimination can also become difficult for some taxa. The many examples of this type of species complex include the *Festuca ovina* aggregate [49], *Dactylis glomerata*[50] and *Knautia arvensis* species groups [51]. The additional and intermittent appearance of facultative or obligatory apomixis, as seen in triploid and tetraploid *Limonium* species [20,21], adds another layer of complexity for species delimitation and diagnosis. In these instances the phenotypic range of taxa can be highly variable, as can their morphological distinctiveness and stability (e.g. [52]). The cumulative effect of these processes is typically manifest in the recognition of a large number of microspecies, with the diagnosis and genetic characterisation of many of the resultant taxa often being highly demanding (e.g. *Limonium*, reviewed in [26]). The publication of a comprehensive revision of *Limonium* species in Southwest Europe by Erben [14] in which 59 species (13 new to science) were fully described allowed for several taxometric studies of geographically related species in this genus to be conducted. For example, various authors used a taxometric approach to study several sexual and agamospermous species of the *L. binervosum* (G. E. Sm.) C. E. Salmon complex from Western Europe [53], and also to examine the closely related *L. vulgare* and *L. humile* species in the British Isles [33]. Morphometric studies have also been used at population level. For instance, some authors studied the taxometric relationships between triploid or aneuploid tetraploid *L. binervosum* agamospermous colonies in the British Isles [42,53]. A similar strategy is applied to describe morphological differentiation patterns between individuals of a *L. dufourii* population from Eastern Spain [54].

In the current study, CDA was applied using ten morphometric traits collected from representative individuals of three diploid and three tetraploid *Limonium* species from Portugal. As previous studies had suggested [14,33,41,42], the collective use of these characters in CDA was sufficient to provide clear morphological differentiation between species at each ploidy level. This differentiation is based primarily on the use of seven morphometric variables, viz: MOBL, MCL, MMBL and MIBL for the first axis and MMBW, MOBW and MIBW on the second. Overall, these analyses not only provided clear separation of all diploid species but also indicated that *L. lanceolatum* and *L. ovalifolium* share a closer phenotypic affinity. Separation of the tetraploid species was also possible but with intermediate individuals blurring the boundaries between *L. vulgare* and *L. dodartii*, and between the latter and *L. multiflorum*. The rather surprising finding, however, lay in the clear phenotypic separation of tetraploid species from the diploids on the basis of the bract and calyx characteristics MCL, MOBL, MMBL and MIBL rather than plant size features more usually associated with ploidy level changes (e.g. see [55]). The relative importance of genetic and epigenetic processes in shaping the observed phenotype structuring among species and ploidy levels in *Limonium* has thus far remained elusive.

Previous works on the analysis of genetic variation and population genetic structure in other *Limonium* species have deployed a wide range of molecular marker systems to infer the importance of genetic structuring in defining interspecies delimitation. There has, nevertheless, been considerable evidence that modest but significant levels of genetic variation does occur within species in the genus. For example, studies on the presumed agamospermous triploid *L. dufourii* have invariably revealed low but substantial inter-individual genetic variation in habitats with significant fragmentation and low population sizes [54,56-58]. Similarly, in diploid *L. dendroides* from Canary Islands, despite radical habitat fragmentation and small population size, some subpopulations of have enough genetic variation to compensate for the influence of drift [59]. However, in plant populations with low genetic variability, epigenetic variation can also act as an important source of potentially adaptive phenotypic variability [11-13]. Nevertheless, the extent and importance of epigenetic variation in natural populations of sexual and agamospermous *Limonium* species is still largely unexplored [9,10,60].

Data generated in the current study unsurprisingly reveals that tetraploids have a higher level of methylation than diploids. In part this may be accounted for the increased genome size, with scope for intergenome heterozysity for methylation marks adding to that expected between homologous loci. There are also additional indirect processes that can contribute to an expected increased incidence in methylation across polyploid species. In newly formed polyploids, genomic duplications from transposons and duplication of regulatory genes, can result in high levels of cytosine methylation on the new portions of the genome [61-63]. Several methylation-based processes have been implicated in the competitive silencing of duplicated gene regions (e.g. [64]), leading to increased methylation relative to the diploid progenitor(s). The same processes also lead to the expectation of differential methylation patterning between the diploid progenitor and the polyploid offspring, partly because of imperfect duplication of genes in the neopolyploid, as was reported for the Waxy gene in *Spartina*[64], but also because of various systems of asymmetric homeolog silencing (e.g. [64]). Given the causal links between methylation and gene regulation [5], there is scope for such changes to influence the phenotype of the polyploids relative to their diploid relatives. Principal coordinate and genetic distance analyses performed in the current study yielded greater separation between diploid and tetraploid taxa when using epigenetic information than when using only genetic information, suggesting that ploidy levels are better separated using epigenetic information than genetic information alone. Intriguingly, this pattern of variation was mirrored by the more pronounced morphological separation between the diploid and tetraploid taxa than that seen between species at either ploidy level. Should this observation apply more broadly to other groups, it implies that epigenetic profiling may provide a useful additional tool to infer ploidy level of preserved specimens.

We found similar trends when making comparisons at the species level. Moreover, the diploid species within *L. ovalifolium* complex have imperfect but reasonable morphological differentiation but genetic co-variation with species identity was relatively modest. Interspecies separation was more strongly enhanced when analysis was focused on the epigenetic variation encompassed in the *Hpa*II profiles, implying that epigenetic patterning (and associated gene silencing) may play a significant role in species separation of the group and so may have some utility for species diagnosis. In both cases, further research would be required to convert these anonymous profiles into Sequence Tagged Site epigenetic markers for robust diagnostic purposes.

Analysis of genetic/epigenetic variability using AMOVA revealed that tetraploid species present lower levels of genetic variability than diploid species. This observation could be most plausibly explained through consideration of their reproductive biology. Several previous studies of the diploids *L. ovalifolium, L. nydeggeri,* and the tetraploids *L. dodartii*, *L. multiflorum* and *L. vulgare* have provided some insights into their primary reproductive strategies [14,31,65]. These and other works were based on the determination of flower dimorphisms linked to a sporophytic self-incompatibility system [14,17,18,28,33,66,67]. In these studies diploids *L. nydeggeri* and *L. ovalifolium* were deemed probable sexual species based on their reproductive characteristics. The same applies to the tetraploid *L. vulgare*[14,65]. Conversely, tetraploids *L. dodartii* and *L. multiflorum* both belonging to the *Limonium binervosum* group, were considered as agamospermous [31,42,53]. Hence, it seems most likely that the lower level of genetic diversity in the putative agamospermous tetraploids could be best explained by their apomictic reproduction mode. In other polyploid apomictic species, such as in *Ranunculus* sp., genetically uniform populations have been similarly observed as a consequence of this mode of reproduction [45]. This finding contrasts with the relatively high level of epigenetic variability among the tetraploids, leading us to speculate that in apomictic polyploid *Limonium* species, the lack of genetic variability caused by the loss of meiotic segregation could be partially compensated by enhanced epigenetic variation. Several authors have suggested similar heritable phenotypic variation due to stable epigenetic variation in the absence of genetic variation (e.g., *Viola cazorlensis*; *Laguncularia racemosa*, and in triploid asexual dandelion lineages (reviewed in [8-10])). Viewed in this context, the results of the present study add *Limonium* to that list.

The present work failed to support any relationship between genetic or epigenetic distance with geographic distance between populations of each species, except for a positive correlation between genetic and geographic distances among *L. nydeggeri*, consistent with regional equilibrium between gene flow and drift [68]. The absence of co-correlation is not unexpected for the apomictic polyploids, and can be explained by restricted gene flow between populations, founder events produced by a limited number of individuals, absence of recombination and spread of single asexual clones within populations [45]. One plausible explanation of these results considered collectively is to propose that genetic information flows between populations but that epigenetic information is mainly induced locally by the environment. Alternatively, it might be that selection pressure on epiloci is higher than on genetic loci, or simply that epiloci are plastic, in the sense that they appear and disappear depending on the environmental cues. Conversely, Mantel test analysis of the correlation between genetic and epigenetic distances between *L. nydeggeri* populations, which presents a regional equilibrium at genetic level, show a strong significant positive correlation between genetic and epigenetic distances. While, genetic and epigenetic distances between *L. multiflorum* populations, which does not present genetic regional equilibrium, showed a weak negative correlation. Again, this might suggest that on the absence of a strong gene flow between populations, environmental conditions exert a higher pressure on the fixation of epigenetic loci that cannot be masked by genetic variability introduced by sexual reproduction.

## Conclusions

Higher correlation was found between morphometric and epigenetic differentiation than between morphometric and genetic differentiation. We therefore, suggest that epigenetic variation might be a driver of the observed phenotypic divergence between the studied taxa through intergenome silencing. We argue that the present work helps to demonstrate the importance of considering phenotypic, genetic and epigenetic variables when seeking to explain the dynamics of complex plant groups that feature hybridization, polyploidy and variable modes of reproductive biology.

## Methods

### Study species

Natural populations of the three diploid species from the *L. ovalifolium* complex (*L. ovalifolium*, *L. nydeggeri*, *L. lanceolatum*) [28,30] and three tetraploids species (*L. dodartii*, *L. multiflorum*, *L. vulgare*) [14,15] were surveyed in the three Portuguese provinces of Estremadura (West), Alentejo (South-West) and Algarve (South). With the exception of *L. lanceolatum* and *L. vulgare* (which grow in salt marshes), all populations vegetated limestone sea-cliffs, in crevices within exposed rocks or on shallow soil above the rock strata and on scree slopes where competition with other species is very low. The locations of all populations were recorded using Global Positioning System. Google Earth 6.0.2 was used for georeferencing and to estimate geographic distances between populations. Geographic mapping of the populations was performed using ArcGIS Desktop 10 (ESRI).

Three leaves per individual were sampled from approximately ten individuals for each population, with a total of 125 plants included in this study. Most individuals were selected from populations within designated SCI(s), namely in SCI Peniche/Sta Cruz (PTCON0056), SCI Sintra/Cascais (PTCON0008) and SCI Costa Sudoeste (PTCON0012) [29]. In all, five *L. nydeggeri* populations were surveyed from Ilha do Baleal (Estremadura: Peniche; SCI PTCON0056), Nossa Srª dos Remédios (Estremadura: Peniche SCI PTCON0056), Cabo Raso (Estremadura: Cascais; SCI PTCON0008), Pontal da Carrapateira (Algarve: Aljezur; SCI PTCON0012), Cabo de São Vicente (Algarve: Vila do Bispo; SCI PTCON0012); two *L. ovalifolium* populations from Cabo de Sagres (Algarve: Vila do Bispo; SCI PTCON0012), and Praia da Luz (Algarve: Lagos); one *L. lanceolatum* population from Vila Nova de Mil Fontes (Alentejo: Odemira; SCI PTCON0012); one *L. dodartii* population from Cabo Sardão (Alentejo: Odemira; SCI PTCON0012); four *Limonium multiflorum* populations from Península da Papoa (Estremadura: Peniche; SCI PTCON0056), Vale dos Frades (Estremadura: Lourinhã; SCI PT PTCON0056), Foz do Lizandro (Estremadura: Mafra; SCI PTCON0056) and Cabo Raso (Estremadura: Cascais; SCI PTCON0008); and one *L. vulgare* population from Vila Nova de Mil Fontes (Alentejo: Odemira; SCI PTCON0012) (see Figure 3; Table 3). Three leaves from plants at the same phenological stage were sampled from all sites during 2010 and kept on silica gel. In this way, variation in DNA methylation profiles attributable to developmental or storage conditions differences was minimized.

### Morphometric analysis

Morphometric analyses were performed in approximately twenty herbarium specimens from each species deposited in the herbaria João de Carvalho e Vasconcellos (LISI; ISA), Portugal. These specimens were previously collected in the same populations selected for MSAP analysis and identified on the basis of species descriptions, diagnostic keys, and locations already described [14], and by comparison with other herbarium specimens present in Portuguese herbaria. The following diagnostic characters were measured: maximum spike length (MSL), maximum number of spikelets per cm (MNSC), maximum number of florets per spikelet (MNFS), maximum outer bract length (MOBL), maximum outer bract width (MOBW), maximum middle bract length (MMBL), maximum middle bract width (MMBW), maximum inner bract length (MIBL), maximum inner bract width (MIBW), and maximum calyx length (MCL). All traits were measured in the lab, after removal of flower parts of each individual. Statistical evaluations were performed with the program SPSS 20 (IBM SPSS, 2010) for Windows. The morphometric variables were tested for deviations from a normal distribution using a Kolmogorov-Smirnov test, and then these variables were log transformed. CDA were conducted to give indication of the degree to which the species were distinguishable from each other and to determine which characters contributed to this discrimination. The box-plots showing the medians and interquartile ranges were produced for each significant character for each species.

### DNA isolation

Three replicate DNA extractions from leaves of each sample were performed from *c*. 0.05 g silica gel dried leaf material using the using the DNeasy 96 Plant Kit (Qiagen, UK) and the Mixer Mill MM 300 (Retsch, Haan, Germany) according to the manufacturers’ instructions. DNA quality and quantity were verified using the nanodrop 2000 spectrophotometer (ThermoScientific, Wilmington, USA). Isolated DNA was diluted in nanopure water to produce working stocks of approximately10 ng μl^-1^.

### Genetic/Epigenetic analyses - MSAP procedure

We used a modification of the MSAP protocol to reveal global variability in CG methylation patterns between samples of the different specimens studied [36]. For each individual, 50 ng of DNA was first digested and ligated using 5U of EcoRI and 1U of *Msp*I or *Hpa*II (New England Biolabs), 0.45 μM EcoRI adaptor, 4.5 μM *Hpa*II adaptor and 1U of T4 DNA ligase (Sigma) in 11 μL total volume of 1X T4 DNA ligase buffer (Sigma), 1 μL of 0.5 M NaCl, supplemented with 0.5 μL at 1 mg/ml of BSA for 2 h at 37°C. The enzymes were then inactivated by heating to 75°C for 15 min. Following restriction and adaptor ligation, there followed two successive rounds of PCR amplification. For pre-selective amplification, 0.3 μL of the restriction/ligation products described above were incubated in 12.5 μL volumes containing 1X Biomix (Bioline, London, UK) with 0.05 μL of PreampEcoRI primer and 0.25 μL Preamp*Hpa*II/*Msp*I (both primers at 10 lM) (Additional file 4) supplemented with 0.1 μL at 1 mg/ml of BSA. PCR conditions were 2 min at 72°C followed by 30 cycles of 94°C for 30 s, 56°C for 30 s and 72°C for 2 min with a final extension step of 10 min at 72°C. Selective PCRs were then performed using 0.3 μL of pre-selective PCR products and the same reagents as deployed for the pre-selective reactions but using FAM labeled selective primers (Additional file 4) Cycling conditions for selective PCR were as follows: 94°C for 2 min, 13 cycles of 94°C for 30 s, 65°C (decreasing by 0.7°C each cycle) for 30 s, and 72°C for 2 min, followed by 24 cycles of 94°C for 30 s, 56°C for 30 s, and 72°C for 2 min, ending with 72°C for 10 min. Initially, eight selective primer combinations (Additional file 4) were evaluated for their ability to detect of inter-specific variation and to generate informative and consistent MSAP profiles using two replicated samples from six different populations (data not shown). Two primer combinations (E1/H1 and E1/H3; Additional file 4) were chosen for the comparative selective amplification.

Fluorescently labelled MSAP products were diluted 1:10 in nanopure sterile water and 1 μL was combined with 1 μL of ROX/HiDi mix (50 μL ROX plus 1 ml of HiDi formamide, Applied Biosystems, USA). Samples were heat-denatured at 95°C for 3–5 min and snap-cooled on ice for 2 min. Samples were fractionated on an ABI PRISM 3100 at 3 kV for 22 s and at 15 kV for 45 min.

### Data analysis

MSAP profiles were analysed using Genemapper 4.0 software (Applera Corporation, Norwalk, Connecticut, USA). For analysis of the genetic/epigenetic variability between samples revealed using MSAP, reproducible product peaks were scored as present (1) or absent (0) to form a raw data matrix. In order to minimize the occurrence of fragment size homoplasy [69] only fragments with lengths between 100 and 500 bp were considered for the analysis. All monomorphic fragments and any fragments present/absent in all but one individual were considered uninformative and removed from all data sets [70]. Reproducibility was estimated calculating the proportion of dimorphic markers between the replicates of the selected samples [70]. For biological error rates, we compared paired MSAP profiles from two leaves of one plant of six populations using each primer combination. Technical reproducibility of the MSAP technique was assessed through the direct comparison of profiles derived from single DNA extractions from ten representative genotypes.

### Analysis of Genetic/Epigenetic Variance

Genetic and epigenetic similarity between tested samples was determined by PCoA [71] based on the MSAP profiles obtained from primer combinations E1/H1 and E1/H3 using GenAlex (v.6.4). Different components of variability were obtained using GenAlex (6.4) software by grouping the samples in two different levels (Populations (i.e. a group of samples) and Regions (i.e. a group of populations). To calculate distances between natural populations, samples from the same natural population restricted with each enzyme were grouped by Populations (this generated two populations from each natural population, one restricted with *Msp*I and one with *Hpa*II) and then at higher level they were grouped into two Regions (all samples restricted with each enzyme). To calculate distances between species, samples from the same taxa restricted with each enzyme were considered and grouped as one Population (this generated two populations from each original species, one restricted with *Msp*I and one with *Hpa*II) and then at higher level they were grouped into two Regions (all samples restricted with each enzyme). We then used AMOVA [72] to evaluate the structure and degree of epigenetic diversity among and between populations, and between species. Pairwise PhiPT [72] comparisons (an analogue of the Fst fixation index, that measures differential connectivity/genetic diversity among populations) between samples restricted with *Msp*I or *Hpa*II was used to infer their overall level of genetic or epigenetic divergence respectively. AMOVA was subsequently calculated using GenAlex (v.6.4) to test the significance of PhiPT between populations and species [73] with the probability of non-differentiation (PhiPT = 0) being estimated over 9,999 permutations. We then calculated genetic diversity estimates (expected heterozygosity, *Hj*) and the actual genetic diversity for each of the groups above, by using Shannon’s index (ShI) [74]. Finally, pairwise genetic or epigenetic and geographical distance (kilometres) matrices were analysed between populations within species and among species by means of a Mantel test. The level of significance was assigned after 1000 permutation tests, as implemented in Genalex 6 [75].

## Abbreviations

AMOVA: Analysis of Molecular Variance; CDA: Canonical Discriminant Analysis; MSL: Maximum spike length; MNSC: Maximum number of spikelets per cm; MNFS: Maximum number of florets per spikelet; MOBL: Maximum outer bract length; MOBW: Maximum outer bract width; MMBL: Maximum middle bract length; MMBW: Maximum middle bract width; MIBL: Maximum inner bract length; MIBW: Maximum inner bract width; MCL: Maximum calyx length; MSAPs: Methylation Sensitive Amplified Polymorphisms; PCoA: Principal Coordinates Euclidean Analysis.

## Competing interests

The authors declare that they have no competing interests.

## Authors’ contribution

ADC designed and coordinated the study. ADC and ASR performed plant sampling. ASR and CMRL contributed to the experimental design and performed the molecular genetic studies by MSAPs. AC and ME realized the morphological measurements. ASR, CMRL and AC processed the raw data and carried out the statistical analysis. CMRL and ADC drafted the manuscript. DES and MJW critically revised the manuscript. All authors read and approved the manuscript.

## Supplementary Material

Additional file 1 **Mean values of morphometric characters in diploid and tetraploid *Limonium *** **species.** Diploid *L. lanceolatum, L. nydeggeri, L. ovalifolium* and tetraploid *L. dodartii, L. multiflorum* and *L. vulgare* species are considered. Click here for file

Additional file 2 **Principal Coordinate Analysis (PCoA) representing genetic and epigenetic variability in diploid and tetraploid *Limonium *** **species.** PCoA was based on presence/absence scores of 347 polymorphic loci obtained from MSAP profiles using isoschizomers *Msp*I (methylation insensitive - red symbols in A and B) or *Hpa*II (methylation sensitive - blue symbols in A and C) as frequent cutters and amplified with primers (E1/H1). The first two coordinates were extracted and plotted against each other. Percentage of the variability shown by each coordinate is indicated between parentheses. Diploid species are represented by solid symbols (*L. lanceolatum*, triangles; *L. nydeggeri*, rhomboids; *L. ovalifolium*, rectangles) and tetraploid species are represented by empty symbols (*L. dodartii*, triangles; *L. multiflorum*, rectangles; *L. vulgare*, circles). Click here for file

Additional file 3 **Estimated genetic (GD) and epigenetic (EpiDG) distances between and within diploid and tetraploid *Limonium *** **species.** Diploid (*L. lanceolatum, L. nydeggeri, L. ovalifolium*) and tetraploid (*L. dodartii, L. multiflorum* and *L. vulgare*) species are considered. Click here for file

Additional file 4 **Oligonucleotides used for MSAP analysis.** Selective nucleotides are indicated as + XYZ in the primer code column. Enzyme column indicates the restriction enzyme site associated with each primer. * FAM labeled selective primers. Click here for file
