# Supplementary figures and images for: Epigenetic rather than genetic factors may explain phenotypic divergence between coastal populations of diploid and tetraploid Limonium spp. (Plumbaginaceae) in Portugal
Source: BMC Plant Biol. 2013 Dec 6;13:205. doi: 10.1186/1471-2229-13-205 (PMC3884021; doi:10.1186/1471-2229-13-205)

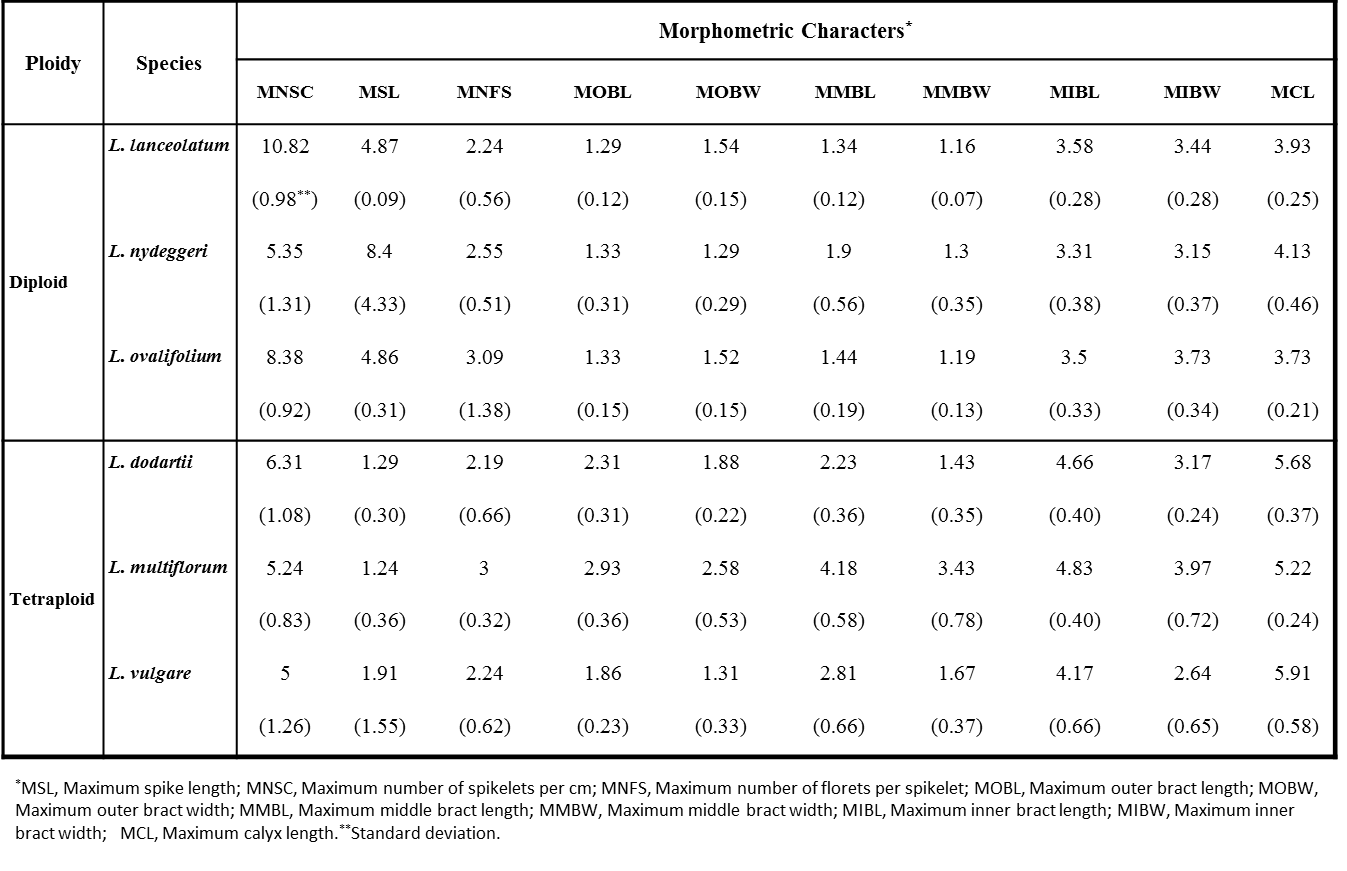

Supplement: Additional file 1 — Mean values of morphometric characters in diploid and tetraploid Limonium species. Diploid L. lanceolatum, L. nydeggeri, L. ovalifolium and tetraploid L. dodartii, L. multiflorum and L. vulgare species are considered. [file 1471-2229-13-205-S1.png]

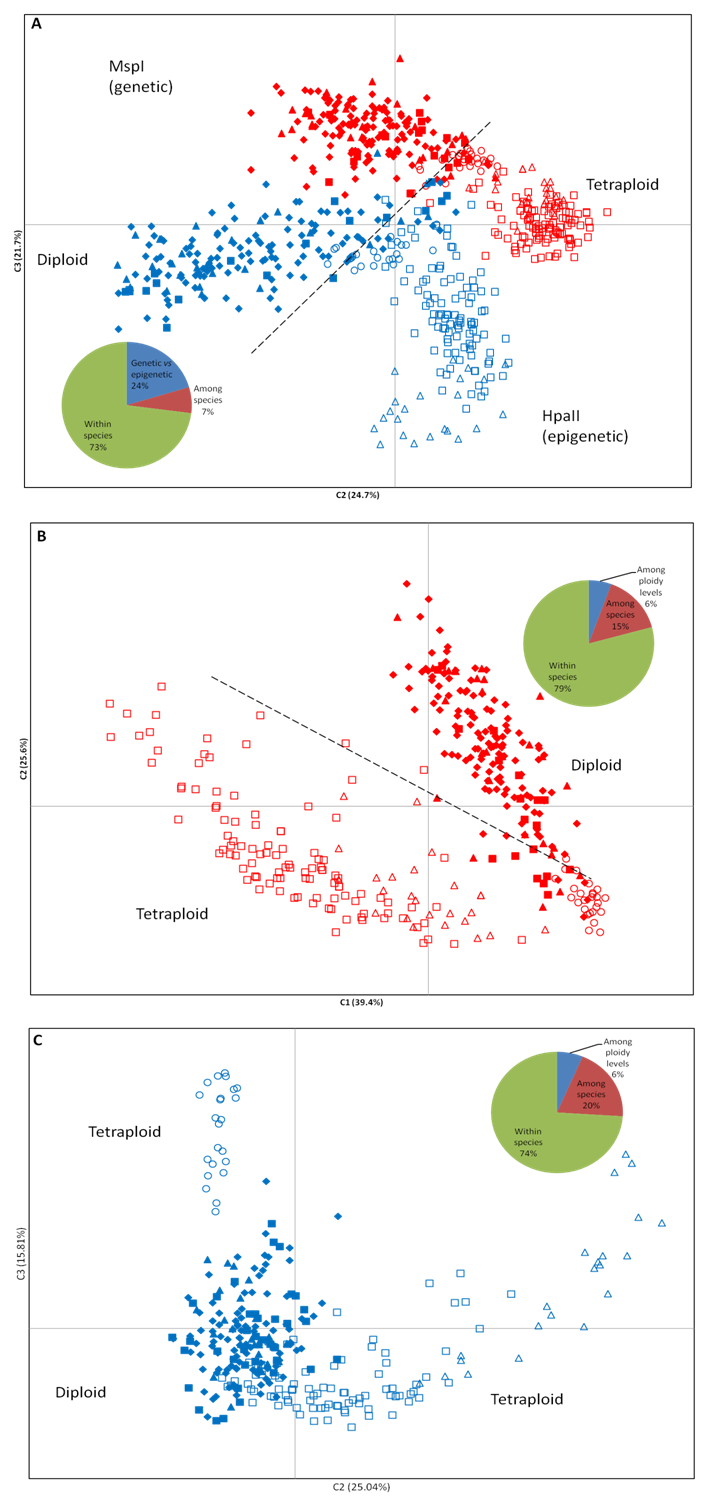

Supplement: Additional file 2 — Principal Coordinate Analysis (PCoA) representing genetic and epigenetic variability in diploid and tetraploid Limonium species. PCoA was based on presence/absence scores of 347 polymorphic loci obtained from MSAP profiles using isoschizomers MspI (methylation insensitive - red symbols in A and B) or HpaII (methylation sensitive - blue symbols in A and C) as frequent cutters and amplified with primers (E1/H1). The first two coordinates were extracted and plotted against each other. Percentage of the variability shown by each coordinate is indicated between parentheses. Diploid species are represented by solid symbols (L. lanceolatum, triangles; L. nydeggeri, rhomboids; L. ovalifolium, rectangles) and tetraploid species are represented by empty symbols (L. dodartii, triangles; L. multiflorum, rectangles; L. vulgare, circles). [file 1471-2229-13-205-S2.png]

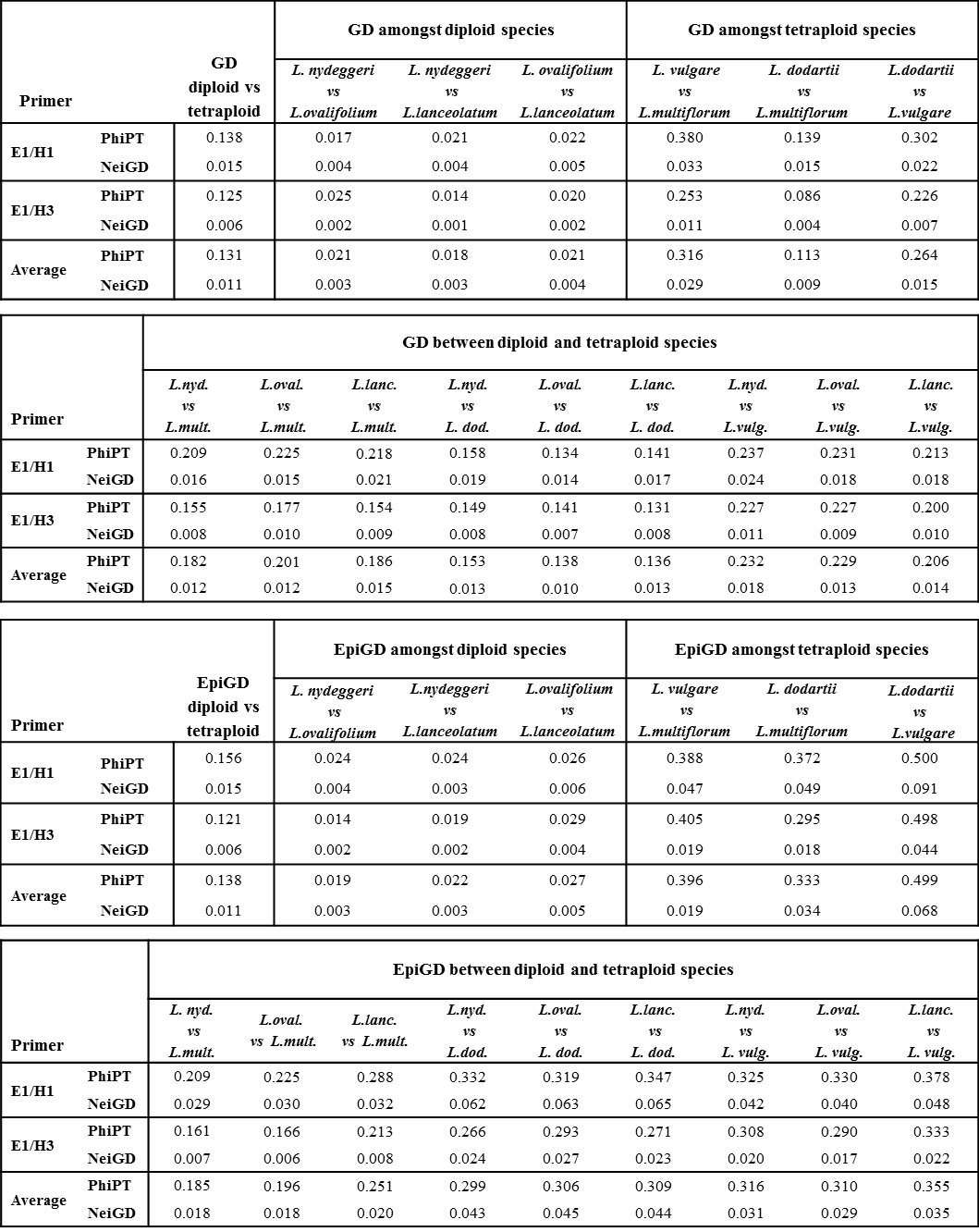

Supplement: Additional file 3 — Estimated genetic (GD) and epigenetic (EpiDG) distances between and within diploid and tetraploid Limonium species. Diploid (L. lanceolatum, L. nydeggeri, L. ovalifolium) and tetraploid (L. dodartii, L. multiflorum and L. vulgare) species are considered. [file 1471-2229-13-205-S3.png]

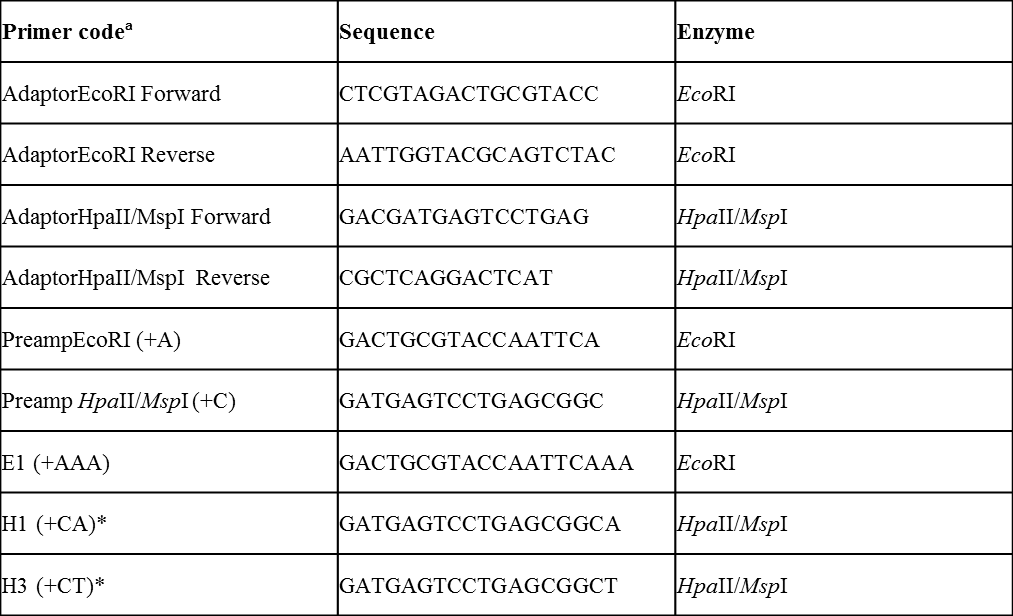

Supplement: Additional file 4 — Oligonucleotides used for MSAP analysis. Selective nucleotides are indicated as + XYZ in the primer code column. Enzyme column indicates the restriction enzyme site associated with each primer. * FAM labeled selective primers. [file 1471-2229-13-205-S4.png]
